# The impact of self-perceived burden, caregiver burden, and dyadic coping on negative emotions in colorectal cancer patient-spousal caregiver dyads: a dyadic analysis

**DOI:** 10.3389/fpsyg.2023.1238924

**Published:** 2023-09-25

**Authors:** Xuan Chen, Zhiming Wang, Junrui Zhou, Chunyan Lin, Huamin Luo, Jie Zhao, Alice Yuen Loke, Qiuping Li

**Affiliations:** ^1^Wuxi School of Medicine, Jiangnan University, Wuxi, Jiangsu Province, China; ^2^Affiliated Hospital of Jiangnan University, Wuxi, China; ^3^School of Nursing, The Hong Kong Polytechnic University, Hung Hom, Kowloon, Hong Kong SAR, China

**Keywords:** colorectal cancer, self-perceived burden, caregiver burden, dyadic coping, anxiety, depression

## Abstract

**Objective:**

To explore the correlation between dyadic coping, self-perceived burden, caregiver burden, and anxiety/depression in colorectal cancer patient-spousal caregiver dyads.

**Methods:**

This study surveyed 200 colorectal cancer patient-spousal caregiver dyads from August 2022 to December 2022. It evaluated self-perceived burden (only for patients), caregiver burden (only for spousal caregivers), dyadic coping, anxiety, and depression. It analyzed data through Pearson’s correlation and the actor–partner interdependence mediation model.

**Results:**

Self-perceived burden and caregiver burden were significantly associated with the anxiety/depression of both individuals in colorectal cancer patient-spousal caregiver dyads; patients’ dyadic coping was associated with self-perceived burden and caregiver burden; caregivers’ dyadic coping was only associated with patients’ dyadic coping and depression. There was an actor–partner mediating effect of self-perceived burden between dyadic coping and anxiety/depression, but there was only a partner-mediating effect of caregiver burden between dyadic coping and anxiety/depression.

**Conclusion:**

This study confirmed the interrelationship between self-perceived burden, caregiver burden, dyadic coping, anxiety, and depression. Self-perceived burden and caregiver burden mediated the relationship between dyadic coping and anxiety/depression in colorectal cancer patient-spousal caregiver dyads. This suggests dynamic interventions for self-perceived burden and caregiver burden can be implemented to improve anxiety/depression in both partners based on maintaining healthy dyadic coping between colorectal cancer patient-spousal caregiver dyads.

## 1. Introduction

Colorectal cancer (CRC) is a major health risk globally and ranks among the top causes of morbidity and mortality among cancer patients (Sung et al., 2021). Despite the increased incidence of CRC, early detection and treatment can lead to a higher survival rate, resulting in an increasing number of CRC survivors (with a 5-year survival rate of 65%; Miller et al., 2019). However, cancer diagnosis and treatment can significantly burden patients and their families, with side effects such as emotional distress and reduced quality of life (Borstelmann et al., 2015). Patients and their caregivers support each other during the prolonged struggle with cancer, and caregivers may play an important role in the clinical pathway of cancer patients, such as providing functional support, emotional support, and decision-making assistance (Cincidda et al., 2022, 2023). In a deepening interactive relationship, patients and their caregivers deepen their impact on each other and may experience a range of burdensome situations because of each other.

For cancer patients, they experience concerns about burdening their caregivers, leading to negative psychological feelings known as self-perceived burden (SPB; Rakic et al., 2018). SPB has been cited as ‘empathetic attentiveness arising from the imbalance of an individual’s disease and caring needs on others, causing guilt, distress, and loss of self-awareness’ (Cousineau et al., 2003; McPherson et al., 2007b). Studies have shown that this empathic concern can cause varying degrees of psychological distress, such as anxiety, depression, and loss of dignity, affecting patients’ quality of life (Akazawa et al., 2010; Dempsey et al., 2012). It has been noted that 19–65% of patients with advanced cancer undergo moderate to extreme SPB (Cousineau et al., 2003; Chochinov et al., 2007; McPherson et al., 2007a). SPB can also cloud the relationship between patients and their caregivers, placing a number of negative effects on them (Rakic et al., 2018; Chen et al., 2023).

Caregivers, especially spousal caregivers (SCs), face a significant caregiving, emotional, and financial burden during the treatment process (Li et al., 2018a). As primary caregivers, spouses are responsible for meeting the daily living, illness care, and emotional support needs of the patient, while also fulfilling additional family and social responsibilities (Li et al., 2018a). However, SCs may neglect their own physical and psychological well-being due to the heavy caregiving load, making them more susceptible to health problems such as fatigue, anxiety, and depression than patients (Janda et al., 2017), thus creating caregiver burden (CB) that results in physical, psychological, emotional, and financial losses associated with providing care (Dang et al., 2008).

Both SPB and CB influenced the mental health of patients and caregivers. A study found that SPB was positively associated with patients’ own anxiety and depression (Kemp et al., 2018). One study noted that SPB can mediate patients’ anxiety (Dempsey et al., 2012). A review concluded that SPB causes severe distress to patients. They described their caregiver-dependent selves as useless, defeated, or out of control, which sometimes left patients feeling isolated or hopeless (Saji et al., 2023). Similarly, studies have demonstrated positive correlations between CB and caregiver anxiety and depression, that is, the increase in CB leads to a rise in anxiety and depression levels (Liu et al., 2017; Hu et al., 2018). This in turn may cause patients to feel guilt and distress about burdening their caregivers (Milbury et al., 2013).

When providing and receiving care, the caregiver and the patient are two related individuals (Berry et al., 2017), and studies have also shown that the physical and mental health of cancer patients and their caregivers are interrelated (Berry et al., 2017; Thompson et al., 2021). Equity theory assumes that individuals try to keep a balance between gaining and giving (Walster et al., 1973). When imbalance occurs, such as when a caregiver provides help that makes the patient feel overly self-beneficial, it can negatively affect both members of the patient–caregiver dyads (McPherson et al., 2007b). In addition, it has been shown that patients’ SPB is associated with greater CB (Kuo et al., 2018). As long as care needs exist for cancer patients, it is difficult to maintain a relatively fair relationship between patients and their caregivers. Both SPB and CB have the potential to occur, with simultaneous impacts on the mental health of both patients and their caregivers. Currently, few studies have used equity theory as a basis for exploring whether SPB and CB have mental effects on both patients and their caregivers from a dyadic perspective. However, the implied equity relationship between SPB and CB exemplifies the inseparable dyadic relationship between patients and their caregivers and may have a dyadic effect on the emotions of both partners.

Therefore, in addition to exploring the impact of SPB and CB on CRC patients and their SCs’ mental health from a dyadic perspective, it is important to understand the coping strategies they use when coping with cancer. Cancer patients’ and caregivers’ coping strategies depend on individual perceptions and cognitions, and different coping strategies may produce different outcomes (Chen et al., 2023). Both patients and their caregivers are in the shadow of cancer, with similar stressful environments (Li et al., 2018a; Petrocchi et al., 2021) and interacting physical and psychological conditions (Janda et al., 2017) that may make cancer patients and their SCs more suited to responding and coping with problems with the dyadic approach. Equity theory helps us to understand how the dyadic relationship between patients and their SCs is created, while the systemic transactional model (STM) proposed by Bodenmann et al. helps us to understand how patients and their SCs respond to a range of stressful problems in a dyadic form. The STM recognizes the interdependence of romantic partners in stress management and adjustment, where both partners are involved in shared problem solving, behavior, and emotion regulation (Bodenmann, 2005; Petrocchi et al., 2021). Dyadic coping (DC) is a type of pressure control from a dyadic perspective. This coping process is regarded as a cyclic bidirectional sequence, affected by mutual dependence between partners (Bodenmann, 2005; Traa et al., 2015). A review indicated that communication, supportive DC, delegated DC, and common DC might reduce stress and promote psychological well-being in cancer patient-spousal caregiver dyads, whereas negative DC was related to worse quality of life and more depressive symptoms (Chen et al., 2021). One study showed that DC was associated with psychological well-being (Facchin et al., 2021); another study also showed that DC was negatively associated with depressive symptoms in cancer patient-spousal caregiver dyads and anxiety symptoms in spouses (Bodschwinna et al., 2021). As previously mentioned, both patients and their SCs are connected to each other. Compared to individual coping, DC could better reflect how patients and their SCs interact with each other throughout the coping process, helping researchers to analyze the outcomes of patients and their SCs from a more holistic perspective. It has been shown through a dyadic approach that DC has an impact on the physical and psychological status of both patients and their caregivers (Li and Loke, 2015; Bodschwinna et al., 2021). However, there is still a lack of research exploring the relationship between DC and SPB or CB.

The goal of this research is to seek the interrelationship between SPB, CB, DC, anxiety, and depression with CRC patients and their SCs through a dyadic approach. SPB, CB, and DC can affect patients and their caregivers’ emotions (Li et al., 2017; Kemp et al., 2018; Bodschwinna et al., 2021), especially anxiety and depression. Therefore, anxiety and depression are studied separately as dependent variables in this study. Although few studies have proved that DC can influence SPB and CB, individual coping can have an effect on SPB and CB (Kuo et al., 2018; Pujol et al., 2018; Kazemi et al., 2021); thus, this study speculates that DC can directly influence SPB and CB and indirectly influence anxiety and depression by affecting SPB and CB. SPB and CB act as mediators and DC as an independent variable in this study. We also hoped to determine whether SPB and CB mediated the impact of DC on anxiety and depression from a dyadic perspective, exploring the direct and indirect relationships between the variables in CRC patients and their SCs. It is also hoped that this study will provide evidence for subsequent dyadic intervention studies. For example, guiding DC to improve SPB and CB may lead to a reduction in anxiety and depression in patients and their SCs.

To verify the relationship between these variables, this study analyzes the results of a cross-sectional survey by using an actor–partner interdependence mediation model (APIMeM). In this model, the actor effect means the effect of an individual’s feature or behavior (e.g., DC) on their own outcomes (e.g., anxiety and depression). The partner effect means the effect of individual’s feature or behavior on their partners’ outcome. This model is composed of six variables as follows: two independent variables (CRC patient-spousal caregiver dyads’ DC), two potential mediator variables (CRC patients’ SPB and SCs’ CB), and two outcome variables (CRC patient-spousal caregiver dyads’ anxiety/depression). To provide a basis for improving anxiety/depression in CRC patient-spousal caregiver dyads through a dyadic approach, this study formulated the following hypotheses: (H1) a correlation exists between DC, SPB, CB, and anxiety/depression; (H2) an actor–partner effect exists between DC, SPB, CB, and anxiety/depression, that is, (i) an actor effect exists between coping and SPB and a partner effect exists between coping and CB in CRC patients; an actor effect exists between coping and CB and a partner effect exists between coping and SPB in spouses; (ii) a partner effect exists between CB and anxiety/depression in CRC patients; a partner effect exists between SPB and anxiety/depression in spouses; (iii) an actor–partner effect exists between coping and anxiety/depression in CRC patients and their SCs; (H3) an actor–partner mediating effect of SPB and CB exists between coping and anxiety/depression in CRC patients and their SCs.

## 2. Methods

The data used in this study were obtained from a cross-sectional survey. It was carried out in a convenience sample at a tertiary hospital in Wuxi, China, in the Department of Medical Oncology and Surgery. Participants included CRC patients and SCs. The study used the STROBE checklist to report results (von Elm et al., 2007).

### 2.1. Participants

The inclusion period was August 2022 to December 2022. Eligibility criteria included the following: (1) CRC patients: diagnosed pathologically with CRC; (2) SCs: spouses who undertake the majority of caregiving duties for CRC patients; (3) CRC patients and SCs both aged over 18 years; (4) both partners have no psychiatric history, can communicate normally, and have civil capacity with knowledge of their condition; (5) patients and SCs can read, understand, express themselves, and write, and can cooperate in completing the questionnaire.

In analyzing data by using SEM, a minimum of 199 couples was estimated to be required to detect a size 0.200 actor or partner effect in the standardized regression coefficients for CRC patients and SCs, ensuring a two-sided type I error of 5% with 80% power (Ackerman and Kenny, 2016). Therefore, this study finally determined the sample size to be 200 couples of CRC patients and SCs.

### 2.2. Measures

This study used a self-designed information scale to extract data from respondents about socio-demographic and cancer-related variables, such as age, gender, and time to disease diagnosis (Table 1). In addition, we investigated the following four variables.

**Table 1:** Characteristics of patients with colorectal cancer and their spouse caregivers.

Characteristics	Patients (*n* = 200) *n* (%)	Spouse caregivers (*n* = 200) *n* (%)
Age years (Mean ± SD)	64.70 ± 0.60	64.20 ± 0.60
31–40	3 (1.50%)	3 (1.50%)
41–50	7 (3.50%)	8 (4.00%)
51–60	54 (27.00%)	62 (31.00%)
61–70	84 (42.00%)	76 (38.00%)
71–80	47 (23.50%)	49 (24.50%)
>80	5 (2.50%)	2 (1.00%)
Gender		
Male	145 (72.50%)	
Female	55 (27.50%)	
Years of marriage		
≤10	4 (2.00%)	
11–20	6 (3.00%)	
21–30	19 (9.50%)	
31–40	80 (40.00%)	
41–50	70 (35.00%)	
51–60	19 (9.50%)	
>60	2 (1.00%)	
Duration		
≤1 month	46 (23.00%)	
1.5–6 months	68 (34.00%)	
6.5–12 months	37 (18.50%)	
12.5–18 months	12 (6.00%)	
18.5–24 months	11 (5.50%)	
>24 months	26 (13.00%)	
Education		
No education	7 (3.50%)	19 (9.50%)
Primary or junior secondary education	130 (65.00%)	126 (63.00%)
High school or post-secondary education	39 (19.50%)	39 (19.50%)
University and above	24 (12.00%)	16 (8.00%)
Religion		
No religion	178 (89.00%)	180 (90.00%)
Buddhism	13 (6.50%)	13 (6.50%)
Christianity	7 (3.50%)	5 (2.50%)
Other	2 (1.00%)	2 (1.00%)
Understanding of the disease		
Completely unknown	21 (10.50%)	27 (13.50%)
Know a little	83 (41.50%)	82 (41.00%)
Basically know	87 (43.50%)	84 (42.00%)
Know very well	9 (4.50%)	7 (3.50%)
Health status		
Good	82 (41.00%)	117 (58.50%)
General	86 (43.00%)	68 (34.00%)
Poor	23 (11.50%)	9 (4.50%)
Very poor	9 (4.50%)	6 (3.00%)
Disease record		
None	87 (43.50%)	97 (48.50%)
Available	113 (56.50%)	103 (51.50%)
Work condition		
Worker	11 (5.50%)	13 (6.50%)
Farmer	23 (11.50%)	15 (7.50%)
Staff	14 (7.00%)	17 (8.50%)
Laid off or unemployed	15 (7.50%)	3 (1.50%)
Sickness or retirement	135 (67.50%)	146 (73.00%)
Other	2 (1.00%)	6 (3.00%)
Monthly household income (¥)		
≤1,000	5 (2.50%)	
1,001-3,000	33 (16.50%)	
3,001–5,000	45 (22.50%)	
>5,000	117 (58.50%)	
Economic pressures		
Heavy	75 (37.50%)	
General	62 (31.00%)	
Low	45 (22.50%)	
None	18 (9.00%)	
With or without stoma pockets		
With stoma pockets	162 (81.00%)	
Without stoma pockets	38 (19.00%)	
Total time spent in care		
<0.5 year		110 (55.00%)
0.5–2 years		58 (29.00%)
2.5–5 years		27 (13.50%)
>5 years		5 (2.50%)

The 29-item Self-Perceived Burden Scale (SPBS) was developed by Tang et al. in China, based on the qualitative interviews with Chinese cancer patients (Tang et al., 2015). It contains six dimensions: care burden, emotional burden, financial burden, social burden, family burden, and psychological burden. A 5-point Likert scale format, with statements varying from “1” (never) to “5” (always), is adopted to lead participants to rate the perceived burden that each item placed on them. The total score is obtained by summing the 29 individual items, and the total score ranged from 30 to 155. A higher score represents more severe SPB. This scale has been validated in cancer patients (Tang et al., 2015); Cronbach’α coefficient was 0.95 (Tang et al., 2015).

The 29-item Caregiver Burden Scale for cancer patients (CBS-CP) surveys family caregivers’ burden in taking care of cancer patients (Li et al., 2017). It contains five dimensions: physical burden, financial burden, psychological burden, social burden, and disease perception burden. Total items in this scale have responses on a 5-point Likert scale from “1” (never) to “5” (always). A score of 29 or less is no burden, 30–58 is a mild burden, 59–87 is a moderate burden, and above 88 is a severe burden. In this scale, the Cronbach’α coefficient was 0.70 ~ 0.96 (Li et al., 2017).

The 37-item Dyadic Coping Inventory (DCI) is set to scale how partners help each other deal with personal stressors and how partners cope together with shared stressors. The items of the scale are all on a 5-point Likert-type scale (1–5). Total scores range from 35 to 175; a score between 111 and 145 is considered normal. The higher the score, the higher the DC rating. The Chinese version of the DCI has been proven to be dependable, with participants’ Cronbach’α coefficient ranging from 0.60 to 0.80 (Xu et al., 2016).

The 14-item Hospital Anxiety and Depression Scale (HADS) is constructed by two 7-item subscales that assess anxiety and depression, respectively (Zhang et al., 2012). All items are marked on a 4-point Likert-type scale (0–3). Subscales of anxiety and depression score vary from 0 to 21. Higher scores in the anxiety and depression subscales indicate anxiety and depression are more severe. Cronbach’s α for subscales of anxiety and depression was 0.87 in cancer patients and 0.85 in family caregivers, respectively (Li et al., 2018c).

Of these, the SPBS is for patients only, and the CBS-CP is for caregivers only. The DCI and HADS are shared scales for patients and SCs. Completing all the scales took participants approximately 10 to 15 min.

### 2.3. Procedures

Before the investigation, the study was approved by the Research Ethics Committee of Jiangnan University (JNU20221201IRB28). The study team communicated with eligible participants after screening according to the inclusion criteria, and gave them written information related to the study to obtain their informed consent. Participants were invited to complete the questionnaires at the hospital when researchers gained informed consent forms from the participants.

### 2.4. Data analysis

SPSS version 26.0 and Amos 24.0 were employed for data processing. The value of *p* < 0.05 was determined as the significance level. Descriptive statistics were calculated to characterize the participants and sum up the data. Pearson correlations were utilized to determine correlations among the study variables for patients and their SCs. To verify whether patients’ and SCs’ DC directly and indirectly affect their own and their partners’ anxiety/depression through the mediation of SPB and CB, the APIMeM was used for examination (Ledermann et al., 2011). In this model, *χ^2^*/degrees of freedom (df), a root mean square error of approximation (RMSEA), a confirmatory fit index (CFI), a goodness of fit index (GFI), and normed fit index (NFI) were used to assess the model fit. A value of less than 3.00 in the *χ^2^/df*, a value of less than 0.08 in the RMSEA, a value of above 0.90 in the GFI, and a value of above 0.95 in the CFI and NFI were considered to be indicative of good model fit (Hopper et al., 2008).

## 3. Results

### 3.1. Descriptive statistics and correlations

The average age of the CRC patients and SCs was 64.70 ± 0.60 (years old) and 64.20 ± 0.60, respectively. The majority of CRC patients were male (72.50%). Approximately 75.00% of couples had been married for 31–50 years. More than half of CRC patients have comorbid non-cancer diseases and more than half of SCs also have non-cancer diseases (s (56.50% for CRC patients and 51.50% for SCs). Over 75.00% of patients had a CRC diagnosis of less than 1 year. Most patients and their spouses were retired (67.50% of CRC patients vs. 73.00% of SCs). CRC couple’s education was mainly primary or junior secondary education (65.00% of patients and 63.00% of SCs). About 90.00% of couples had no religious affiliation (89.00% of CRC patients and 90.00% of SCs). More than half of couples had a monthly household income greater than ¥5,000 (58.50%), but more than half of patients felt there was an average (31.00%) to heavy (37.50%) financial burden. More than half of spouses had cared for their patients for less than 6 months (55.00%). More details are shown in Table 1.

Table 2 shows the correlations among the variables for CRC patients and SCs. For CRC patients, DC was positively correlated with their own SPB (*r* = 0.39, *p* < 0.01); it had a positive correlation with the CB (*r* = 0.15, *p* < 0.01) and DC (*r* = 0.51, *p* < 0.01) of SCs. Unexpectedly, patients’ DC was not related to both CRC patients and SCs’ anxiety and depression. SPB was positively correlated with their own anxiety (*r* = 0.55, *p* < 0.01) and depression (*r* = 0.44, *p* < 0.01); it also had a positive association with SCs’ CB (*r* = 0.50, *p* < 0.01), anxiety (*r* = 0.43, *p* < 0.01), and depression (*r* = 0.41, *p* < 0.01). For SCs, DC was only positively correlated with patients’ DC and had adverse correlation with patients’ depression (*r* = −0.21, *p* < 0.01). It was not related to patients’ SPB and anxiety, it was also irrelevant to their own CB, anxiety, and depression. CB was positively correlated with patients’ SPB, DC, anxiety (*r* = 0.43, *p* < 0.01), and depression (*r* = 0.38, *p* < 0.01); it was also positively correlated with their own anxiety (*r* = 0.61, *p* < 0.01) and depression (*r* = 0.57, *p* < 0.01).

**Table 2:** Pearson’ s correlations and descriptive statistics of the study variables (*n* = 200).

Variables***	M	SD	1	2	3	4	5	6	7	8
SPBS	65.58	21.47	1							
CBS-CP	20.56	13.17	0.50**	1						
DCI-P	95.22	13.82	0.39**	0.15*	1					
DCI-SC	90.14	12.85	0.08	0.09	0.51**	1				
Anxiety-P	7.11	3.87	0.55**	0.43**	0.05	−0.12	1			
Depression-P	8.46	4.20	0.44**	0.38**	−0.13	−0.21**	0.85**	1		
Anxiety-SC	5.84	4.20	0.43**	0.61**	0.13	0.00	0.45**	0.42**	1	
Depression-SC	6.96	4.59	0.41**	0.57**	0.11	−0.07	0.43**	0.42**	0.92**	1

M, mean; SD, standard deviation. *Correlation is significant at the 0.05 level (2-tailed); **Correlation is significant at the 0.01 level (2-tailed); ***SPB = Self-Perceived Burden Scale. *CBS-CP*, Caregiver Burden Scale for Cancer Patients; DCI, Dyadic Coping Inventory; P, patient; SC, spousal caregiver.

### 3.2. Mediation analysis with actor–partner interdependence mediation model

**Figure 1:**
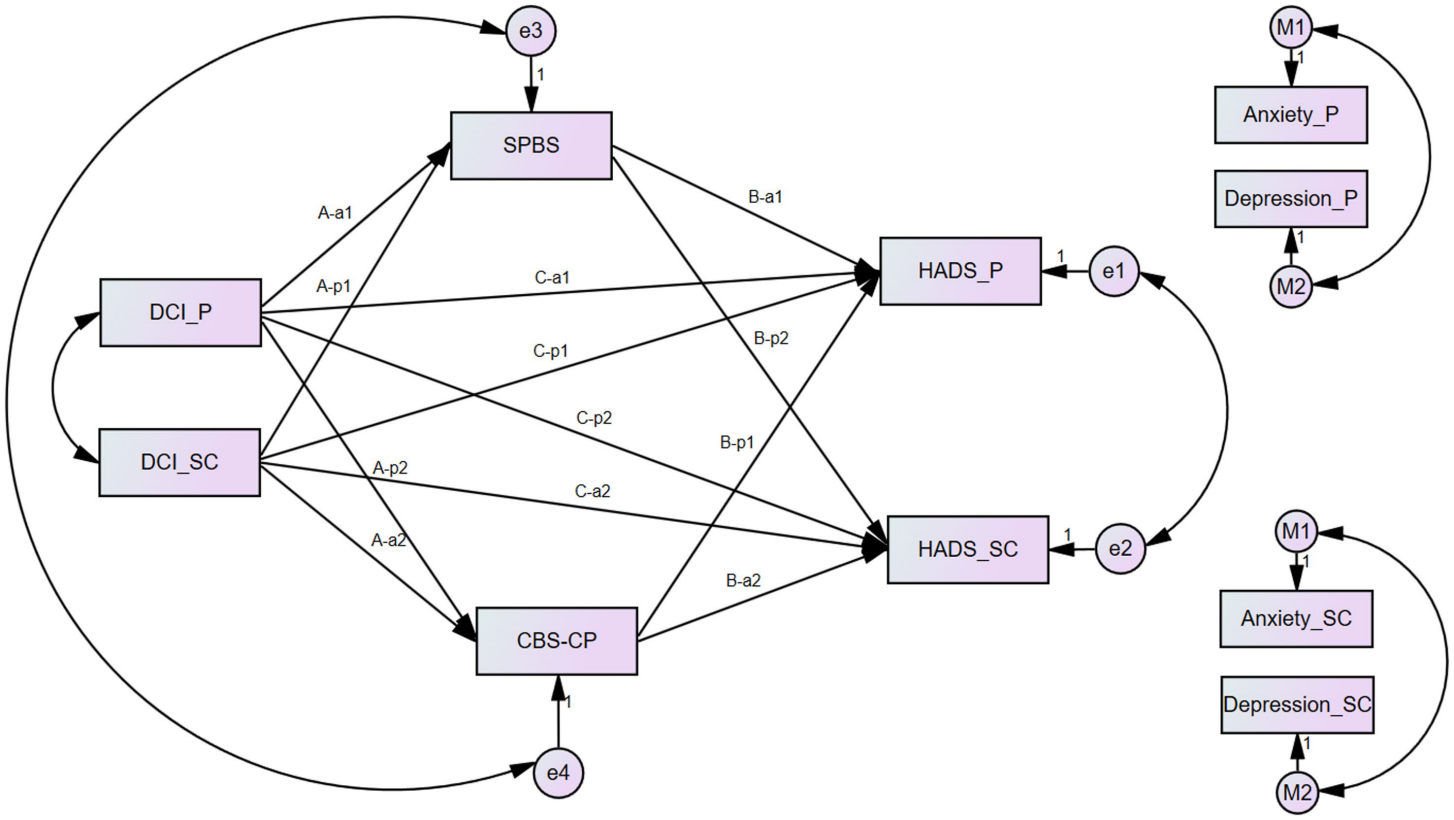
Actor–partner independence mediation model of dyadic coping, self-perceived burden, caregiver burden, and anxiety/depression. Note: HADS were replaced by the M1 and M2, respectively, in two different models as indicated in the figures. A-a1, A-a2, B-a1, B-a2, C-a1, and C-a2 stand for Actor effects. A-p1, A-p2, B-a1, B-p2, C-p1, and C-p2 stand for Partner effects. P, patients, SC, spousal caregivers.

Figure 1 shows the APIMeM of DC, SPB, CB, and anxiety/depression. As shown, DC through SPB and CB may directly or indirectly impact the HADS as actor effects (A-a1, A-a2, B-a1, B-a2, C-a1, and C-a2) and/or as partner effects (A-p1, A-p2, B-a1, B-p2, C-p1, and C-p2) in the two models, respectively. Anxiety and depression scores would be brought into the model separately, resulting in two sub-models (anxiety was carried into sub-model 1, depression into sub-model 2).

In the Anxiety model (see Supplementary Figure S1), the model did not present a good fit: *χ^2^/df = 6.27, p* < *0.001, RMSEA = 0.16, CFI = 0.91, GFI = 0.94, NFI = 0.89*. After removing the non-significant paths and taking into account modification indices, the final model (see Supplementary Figure S2) obtained had a satisfactory fit to the data: *χ^2^/df = 1.02, RMSEA = 0.01, CFI = 1.00, GFI = 0.99, NFI = 0.99*. All the paths were statistically significant. Table 3 presents the related indices for the final model.

**Table 3:** Standardized path coefficients and fit statistics of two adjusted models.

Indicates	Estimate	*S.E.*	*P*	Standard estimate
Anxiety				
SPBS ← DCI_P (A-a1)	0.74	0.11	***	0.47
SPBS ← DCI_SC (A-p1)	−0.29	0.11	***	−0.17
CBSCP ← DCI_P (A-p2)	0.15	0.07	**	0.15
Anxiety_P ← DCI_P (C-a1)	−0.05	0.02	***	−0.18
Anxiety_P ← SPBS (B-a1)	0.09	0.01	***	0.52
Anxiety_SC ← CBSCP (B-a2)	0.17	0.02	***	0.52
Anxiety_P ← CBSCP (B-p1)	0.06	0.02	***	0.20
Anxiety_SC ← SPBS (B-p2)	0.03	0.01	***	0.17
Depression				
SPBS ← DCI_P (A-a1)	0.74	0.11	***	0.47
SPBS ← DCI_SC (A-p1)	−0.29	0.11	***	−0.17
CBSCP ← DCI_P (A-p2)	0.15	0.07	**	0.15
Depression_P ← DCI_P (C-a1)	−0.11	0.02	***	−0.35
Depression_SC ← DCI_SC (C-a2)	−0.04	0.02	***	−0.12
Depression_P ← SPBS (B-a1)	0.09	0.01	***	0.48
Depression_SC ← CBSCP (B-a2)	0.17	0.02	***	0.50
Depression_P ← CBSCP (B-p1)	0.06	0.02	***	0.20
Depression_SC ← SPBS (B-p2)	0.04	0.01	***	0.18

***Correlation is significant at the 0.01 level (2-tailed); **Correlation is significant at the 0.05 level (2-tailed); SPB, Self-Perceived Burden Scale; CBSCP, Caregiver Burden Scale for Cancer Patients; DCI, Dyadic Coping Inventory; P, patients; SC, spousal caregivers.

In the Depression model (see Supplementary Figure S1), the model did not have satisfactory fit to the data: *χ2/df = 6.93, p < 0.001, RMSEA = 0.17, CFI = 0.89, GFI = 0.94, NFI = 0.88*. After removing the non-significant paths and taking into account modification indices, the final model (see Supplementary Figure S2) obtained had a better fit to the data: *χ2/df = 1.23, RMSEA = 0.03, CFI = 1.00, GFI = 0.99, NFI = 0.99.* All the paths were statistically significant. Table 3 presents the related indices for the final model.

Thus, there was an actor effect between DC and SPB and a partner effect between DC and CB in CRC patients, and a partner effect existed between DC and SPB in spouses, but there was not an actor effect between DC and CB. Meanwhile, the results proved hypothesis 2, in that there was an actor effect between SPB and anxiety/depression in CRC patients and a partner effect between SPB and anxiety/depression in SCs; there was also an actor effect between CB and anxiety/depression in SCs and a partner effect in patients. However, in the Anxiety model, DC only had an actor effect on patients’ anxiety/depression; in the Depression model, DC had an actor effect on both patients’ and SCs’ anxiety/depression, but without a partner effect. It also proved that there was an actor–partner mediating effect of SPB between DC and anxiety/depression, but there was only a partner-mediating effect of CB between DC and anxiety/depression.

## 4. Discussion and conclusion

This study intended to scrutinize the relationships between the variables of SPB, CB, DC, anxiety, and depression, and to ascertain whether there is an actor–partner mediating effect of SPB and CB that exists between coping and anxiety/depression in CRC patients and SCs. The results of the study verified most of our hypotheses. The discussion that follows will focus on two corresponding aspects based on the hypotheses and results.

### 4.1. Correlations between self-perceived burden, caregiver burden, dyadic coping, and dyads’ anxiety/depression

By testing the correlation between the variables of CRC patients and SCs, SPB was significantly associated with CB; SPB and CB were also significantly associated with the anxiety and depression of both individuals in CRC patient-spousal caregiver dyads, which was similar to previous studies (Hu et al., 2018; Kemp et al., 2018). Although this study verified that patients’ DC was associated with SPB and CB, unlike previous studies, patients’ DC was not significantly correlated with anxiety/depression in CRC patient-spousal caregiver dyads; meanwhile, caregivers’ DC was only associated with patients’ DC and depression. This suggests that patients’ DC may not directly improve their own and SCs’ anxiety and depression. Whereas, SCs’ starting point for DC may be to help patients to gain a better outcome. Therefore, SCs’ DC ability can affect patients’ DC ability to a certain extent and improve patients’ depression based on this.

### 4.2. The actor–partner effect between dyadic coping, self-perceived burden, caregiver burden, and anxiety/depression

In the APIMeM, there was an actor effect between CRC patients’ DC and SPB and a partner effect between CRC patients’ DC and CB, and both of them were positive effects. A review found that DC may reduce couples’ burden and promote mutual mental health (Chen et al., 2021). However, this study found a higher degree of CRC patients’ DC exacerbates their own SPB, as well as SCs’ CB. It might be that DC requires patients and SCs to cope together as a unit in their journey of against CRC, and higher scores of DC may require SCs to give more energy and time to match the patients’ rhythm, which potentially increases CB. Simultaneously, excessive caregiver efforts may also increase patients’ guilt, which affect SPB. It was also found that DC from SCs’ perspective only had a negative partner effect on SPB. A similar result was obtained in one previous study (Kazemi et al., 2021); good DC status was effective in improving SPB and also in promoting communication and understanding between couples. It was surprising that SCs perceived no effect of DC on their own CB. Maybe from SCs’ perspective, the main purpose of DC is to improve patients’ physical and psychological status and it does not produce a significant change in caregivers themselves. It is interesting to consider that DC from CRC patients’ and SCs’ perspectives have very different effects on SPB and CB. Although DC is a form of stress management from a dyadic perspective, DC is also affected by the degree of mutual communication and interdependence between couples (Bodenmann, 2005; Traa et al., 2015). This implies that communication between Chinese CRC patient-spousal caregiver dyads may be lacking, leaving no overall agreement between couples’ coping attitudes. As the results demonstrate, inconsistent coping attitudes may lead patients to believe that DC exacerbates CB and SPB. They are likely to reduce DC, even though positive coping is effective in improving their own physical and psychological conditions.

Previous studies have demonstrated that SPB can affect patients’ negative emotions (Akazawa et al., 2010; Dempsey et al., 2012) and CB can affect SCs’ negative emotions (Dang et al., 2008). Through the model validation, this study not only supported previous studies but also confirmed our hypothesis. There are positive actor–partner effects of SPB and CB on anxiety and depression, respectively. This suggests to us that the exacerbation of SPB and CB affects not only oneself but also one’s partner’s anxiety and depression, which is consistent with one previous study yielding results (Milbury et al., 2013). It also demonstrates the need for a dyadic intervention in the clinical setting. However, most studies have conducted interventions for either SPB or CB alone (Li et al., 2015; An et al., 2020; Treanor, 2020), and few have considered the relationship between SPB and CB simultaneously and their impact on patient–caregiver’s dyadic outcomes. There have been a number of studies that show the feasibility and effectiveness of dyadic intervention (Li and Loke, 2015; Bodschwinna et al., 2021; Ferraris et al., 2022), which suggests that we can learn from these examples of dyadic interventions for SPB and CB.

In addition, this study verified whether there was an actor–partner effect of DC on anxiety and depression. In the Anxiety model, there was a negative actor effect of DC on anxiety from the patient perspective only; in the Depression model, there was a negative actor effect of DC on depression, which was consistent with a previous study (Bodschwinna et al., 2021). Meanwhile, there was no partner effect of DC on anxiety/depression. The existence of an actor effect indicated that there was an impact of DC on one’s own anxiety/depression, but coping on a dyadic basis did not have an impact on the partner’s anxiety/depression. One possible reason is that DC affects patients themselves quickly, whereas it may take time to affect partners, which may suggest that we need to conduct longitudinal studies to explore the duration of DC effects on patients and on partners.

### 4.3. Self-perceived burden and caregiver burden act as mediators for dyadic coping and anxiety/depression

The results showed an actor–partner mediated effect of SPB between DC and anxiety/depression. However, if SPB is lessened, it will improve the anxiety/depression of CRC patient-spousal caregiver dyads based on reducing patients’ DC and increasing SCs’ DC. Patients’ and SCs’ DC have different effects on SPB; this may suggest that interventions for SPB need to be carried out not only to improve SPB but also to observe whether there is a dynamic balance between patients’ DC and SCs’ DC. The results also showed a partner-mediated effect of CB between DC and anxiety/depression. This means that improved CB can improve anxiety/depression on both sides on the basis of improved patients’ DC.

A study showed that the occurrence of cancer can affect a family’s emotional wellness and quality of life (Borstelmann et al., 2015), while negative emotions such as anxiety and depression can also affect the quality of life of patients and their caregivers (Li et al., 2018b). Perhaps this suggests that clinical nursing staff can improve anxiety and depression directly or indirectly by intervening with DC, SPB, and CB, and then improve the quality of life for both patients and SCs, enabling better outcomes for a family coping with a cancer diagnosis.

### 4.4. Limitation

Firstly, cross-sectional studies make it difficult for outcome variables to reflect the trajectory of progression. Furthermore, the participants’ specific social and cultural backgrounds, e.g., Chinese CRC patient-spousal caregiver dyads, restricted the applicability of the outcomes to other populations from other cultural backgrounds. Third, the current sample size may have biased the results. Future large sample longitudinal studies with participants from different cultural backgrounds are needed.

### 4.5. Implications for practice

Although limitations exist, this study showed that the associations between coping and anxiety/depression were mediated mainly by SPB and CB, which was important for improving both CRC patients and SCs’ anxiety/depression. For medical and nursing staff within cancer practice, it is necessary to intervene CRC patient-spousal caregiver dyads as a unit in a dyadic way. In caring for CRC patient-spousal caregiver dyads, dyadic-based interventions, involving elements such as alleviating dyads’ burden (including SPB and CB) and cultivating positive dyadic active coping mutual relationships, are highly recommended to improve anxiety and depression for both partners. As the results of this study showed a significant effect of patients’ SPB on both patients’ and SCs’ anxiety and depression, interventions could be more focused on improving patients’ SPB and encouraging patients to cope positively with SPB, which would indirectly improve SCs’ anxiety and depression. The differential effect of DC on SPB and CB also suggests that individualized interventions need to be developed to improve DC capacity (Chen et al., 2021), particularly SCs’ DC capacity, which can improve patients’ SPB.

### 4.6. Conclusion

In this study, we confirmed the interrelationship between SPB, CB, DC, anxiety, and depression. We also verified the existence of actor–partner effects among the variables and proved an actor–partner mediating effect of SPB between DC and anxiety/depression and a partner-mediating effect of CB between DC and anxiety/depression. This suggests that we can dynamically intervene with SPB and CB to improve anxiety and depression in both partners under the premise of maintaining healthy DC between CRC patient-spousal caregiver dyads.

## Data availability statement

The raw data supporting the conclusions of this article will be made available by the authors, without undue reservation.

## Author contributions

XC: conceptualization, formal analysis, methodology, investigation, and writing—original draft. ZW and JuZ: formal analysis, methodology, investigation, and writing—original draft. CL, HL, and JiZ: methodology, investigation, resources, and supervision. AL and QL: supervision and writing—review and editing. All authors contributed to the article and approved the submitted version.

## Funding

This work was supported by the National Natural Science Foundation of China [grant number 82172844]. The funders had no role in study design, data collection and analysis, decision to publish, or preparation of the manuscript.

## Conflict of interest

The authors declare that the research was conducted in the absence of any commercial or financial relationships that could be construed as a potential conflict of interest.

## Publisher’s note

All claims expressed in this article are solely those of the authors and do not necessarily represent those of their affiliated organizations, or those of the publisher, the editors and the reviewers. Any product that may be evaluated in this article, or claim that may be made by its manufacturer, is not guaranteed or endorsed by the publisher.
