# Supplementary material for: The impact of self-perceived burden, caregiver burden, and dyadic coping on negative emotions in colorectal cancer patient-spousal caregiver dyads: a dyadic analysis
Source: Front Psychol. 2023 Sep 25;14:1238924. doi: 10.3389/fpsyg.2023.1238924 (PMC10561240; doi:10.3389/fpsyg.2023.1238924)
Supplement: Supplementary file 1 [file Data_Sheet_1.docx]

Supplementary Material

The impact of Self-perceived burden, Caregiver burden, Dyadic coping on negative emotions in colorectal cancer couples: A dyadic analysis

Xuan Chen, Zhiming Wang, Junrui Zhou, Chunyan Lin, Huamin Luo, Jie Zhao, Qiuping Li^*^

## Supplementary Figure

**Supplementary Figure 1.** Supporting figures of Sub-model 1-2 for testing the assumption that Dyadic coping directly or indirectly influences the negative emotions (anxiety and depression) of CRC patient-caregiver dyads through Self-perceived burden and caregiver burden

1.
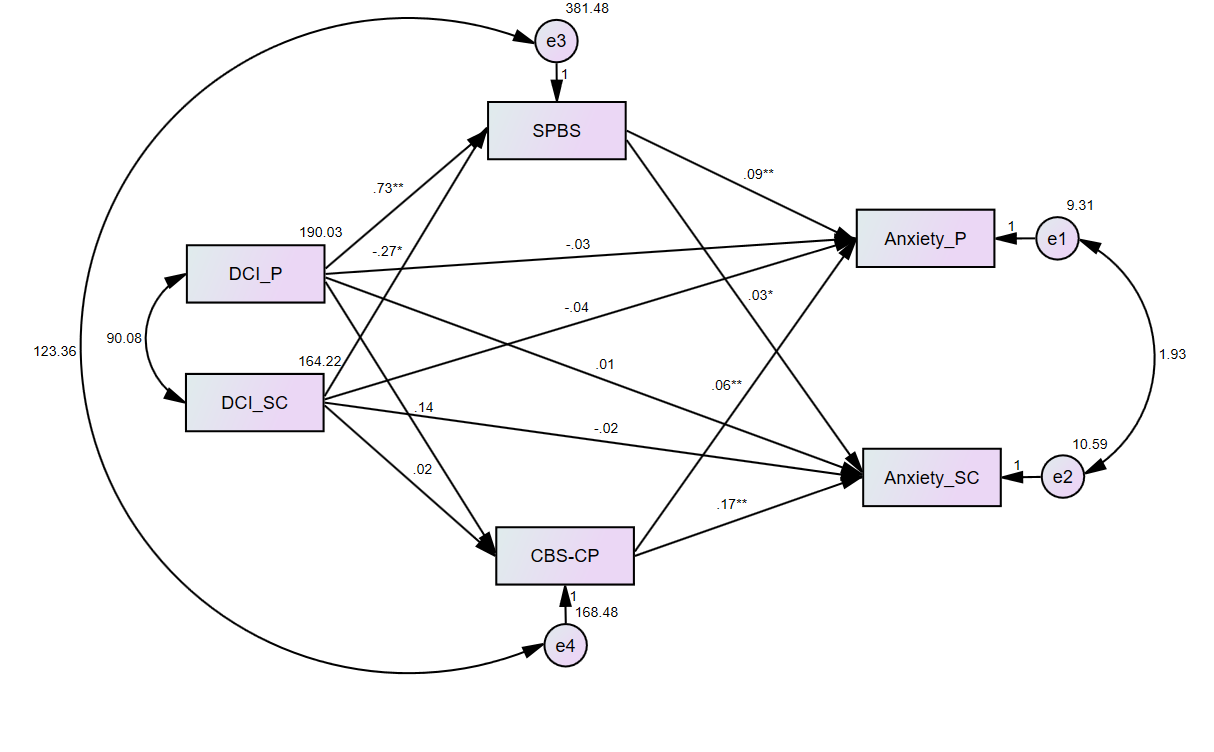
Sub-model 1, Anxiety

**P<0.05; **P<0.01*

2.Sub-model 2, Depression


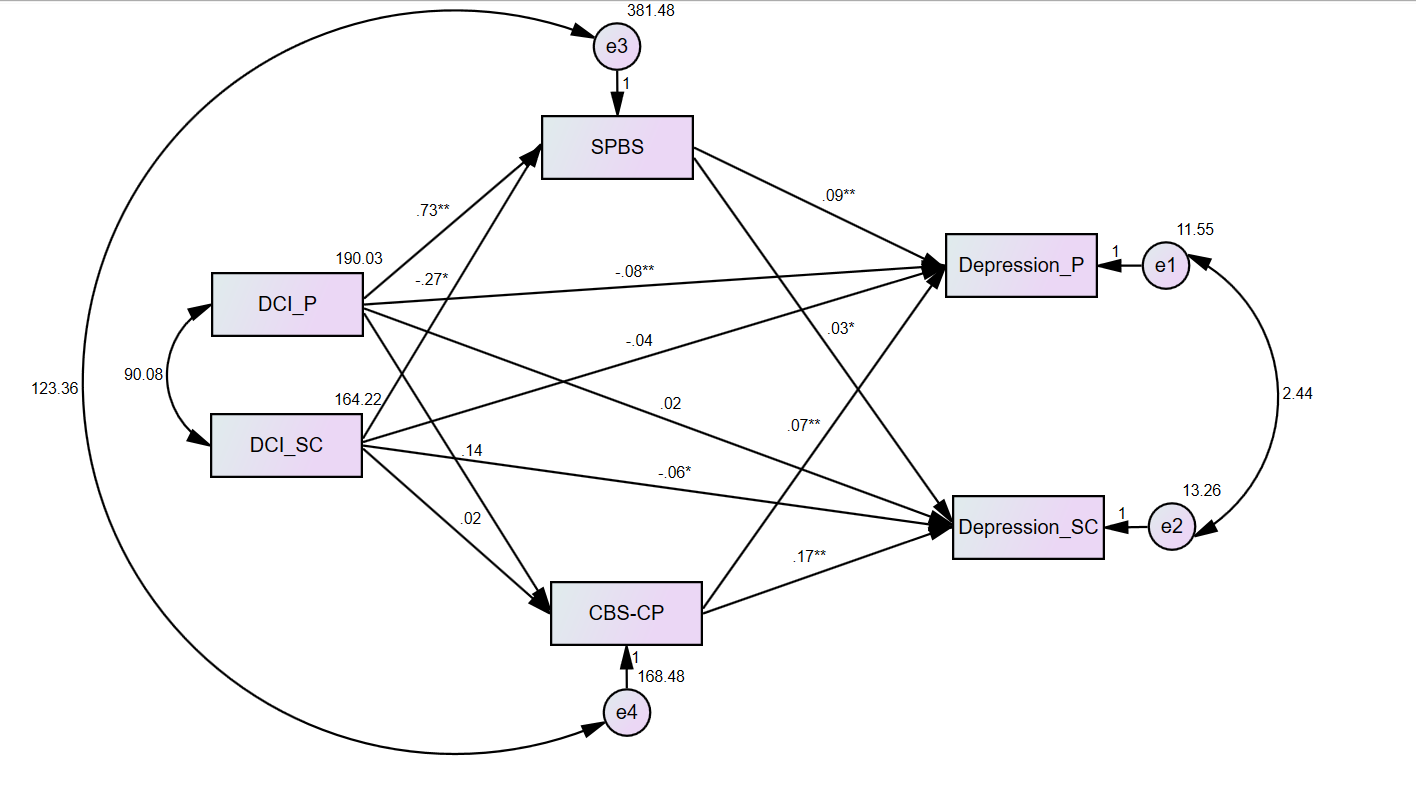


**P<0.05; **P<0.01*

**Supplementary Figure 2.** Supporting figures of the final Sub-model 1-2

1. Sub-model 1, Anxiety


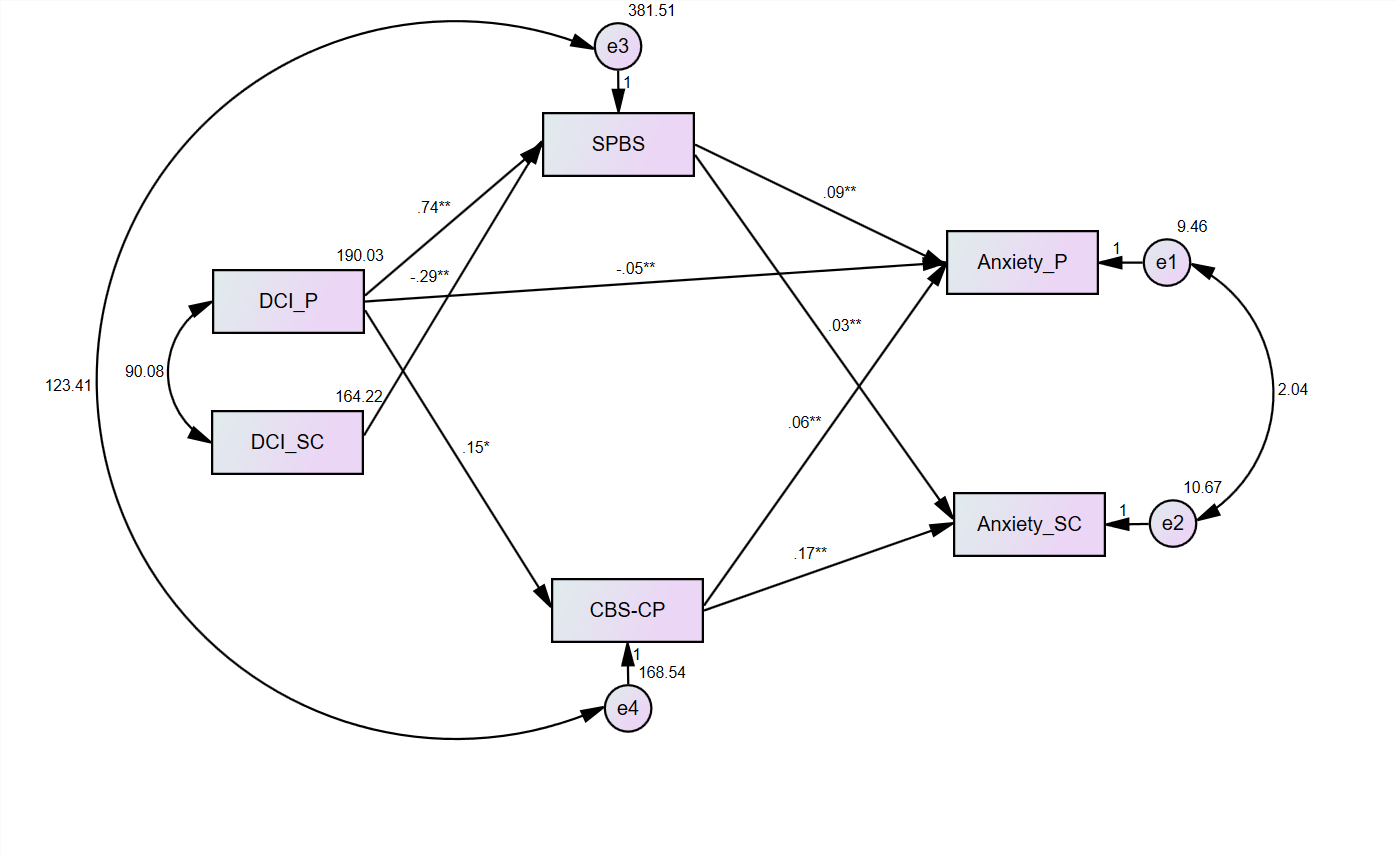


**P<0.05; **P<0.01*

2. Sub-model 2, Depression


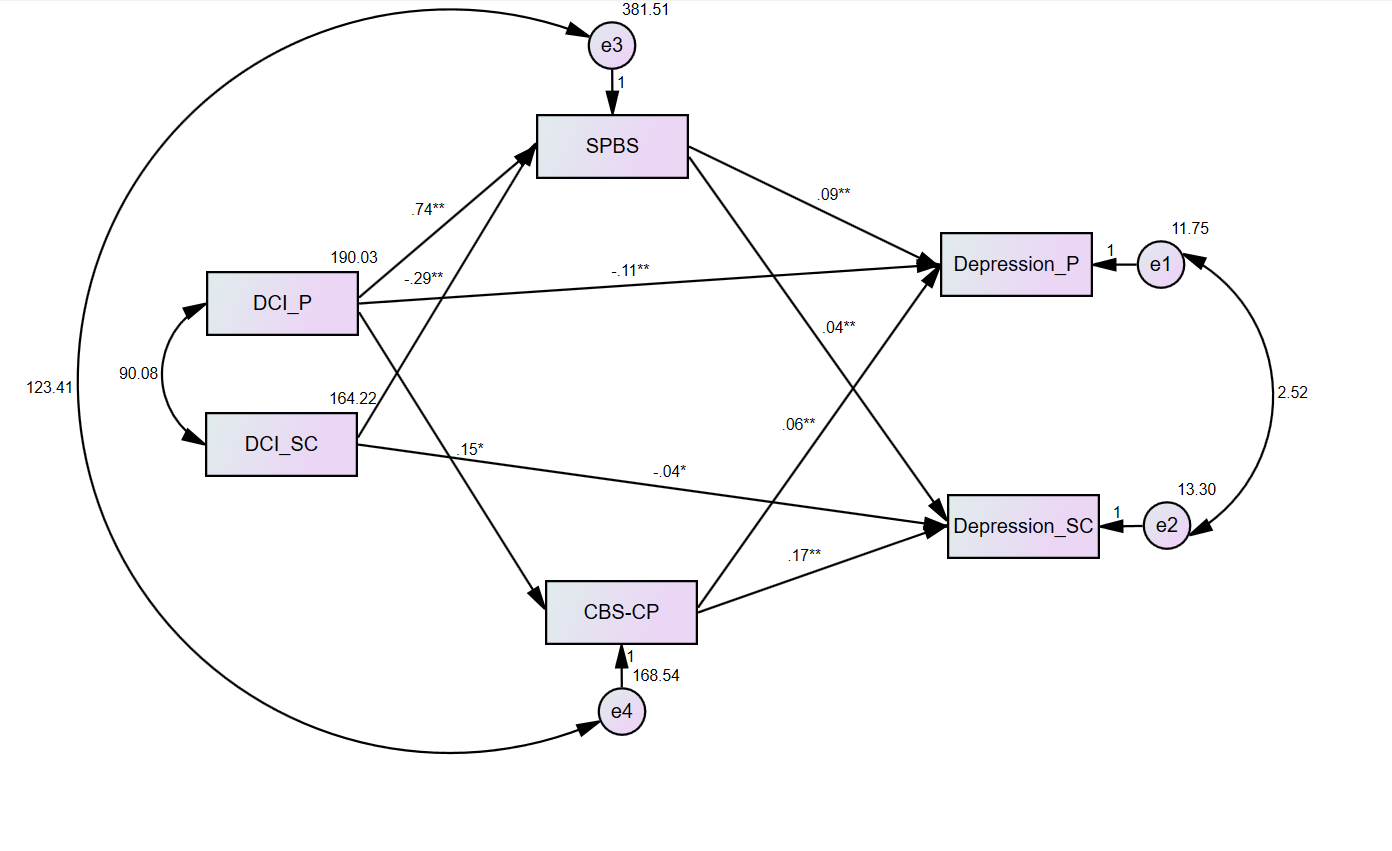


**P<0.05; **P<0.01*
